# Supplementary material for: Cyromazine Effects the Reproduction of Drosophila by Decreasing the Number of Germ Cells in the Female Adult Ovary
Source: Insects. 2022 Apr 27;13(5):414. doi: 10.3390/insects13050414 (PMC9144682; doi:10.3390/insects13050414)
Supplement: Supplementary file 1 [file insects-13-00414-s001.zip › insects-1674480-supplementary.pdf]

Table S1: Primer's sequence

Name	Primer Sequences (*5'-3'*)
EcR	caggaggaccagatcacgtt
	tcacaatggcagtgagaagc
usp	aataaagtgcgctgctccat
	cggtttcttcttgctcgttc
E75B	tcatcactcgaaccctaccc
	accactaccgtggcttcatc
E78	cggaggaactgaccagagag
	caaccagacctcgaagaagc
sad	aaggagcgagctaccaatga
	gctgctcaaagtgtgatgga
spok	ttacggttttggcacattga
	tgtcgccgagctaaatttct
kr-h1	ttcagcgtgaaggagaacct
	ccggactggatgaatgtctt
nvd	tggacaacgaaaaatggtca
	actagatcttcgcccaagca
tpr2	ttcggccatacaaaacacaa
	tccactgctttttcgctttt
Vkg	tggacgctgcaactactacg
	accatctgtgaaaccggaag
Hrb27C	cagcaaaagtccaagtgcaa
	ttttgtcaacccgtcatcaa
CycE	agctaccaggaacctcagca
	gaggcaaccgatgacagatt
